# Steady‐state activation and modulation of the synaptic‐type *α*1*β*2*γ*2L GABA_A_ receptor by combinations of physiological and clinical ligands

**DOI:** 10.14814/phy2.14230

**Published:** 2019-09-23

**Authors:** Allison L. Germann, Spencer R. Pierce, Thomas C. Senneff, Ariel B. Burbridge, Joe Henry Steinbach, Gustav Akk

**Affiliations:** ^1^ Department of Anesthesiology Washington University School of Medicine St. Louis Missouri; ^2^ Taylor Family Institute for Innovative Psychiatric Research Washington University School of Medicine St. Louis Missouri

**Keywords:** Anesthetics, electrophysiology, GABA_A_ receptors, neurosteroids

## Abstract

The synaptic *α*1*β*2*γ*2 GABA_A_ receptor is activated phasically by presynaptically released GABA. The receptor is considered to be inactive between synaptic events when exposed to ambient GABA because of its low resting affinity to the transmitter. We tested the hypothesis that a combination of physiological and/or clinical positive allosteric modulators of the GABA_A_ receptor with ambient GABA generates measurable steady‐state activity. Recombinant *α*1*β*2*γ*2L GABA_A_ receptors were expressed in *Xenopus* oocytes and activated by combinations of low concentrations of orthosteric (GABA, taurine) and allosteric (the steroid allopregnanolone, the anesthetic propofol) agonists, in the absence and presence of the inhibitory steroid pregnenolone sulfate. Steady‐state activity was analyzed using the three‐state cyclic Resting‐Active‐Desensitized model. We estimate that the steady‐state open probability of the synaptic *α*1*β*2*γ*2L GABA_A_ receptor in the presence of ambient GABA (1 *μ*mol/L), taurine (10 *μ*mol/L), and physiological levels of allopregnanolone (0.01 *μ*mol/L) and pregnenolone sulfate (0.1 *μ*mol/L) is 0.008. Coapplication of a clinical concentration of propofol (1 *μ*mol/L) increases the steady‐state open probability to 0.03. Comparison of total charge transfer for phasic and tonic activity indicates that steady‐state activity can contribute strongly (~20 to >99%) to integrated activity from the synaptic GABA_A_ receptor.

## Introduction

The *α*1*β*2*γ*2 GABA_A_ receptors in the central nervous system are concentrated in the postsynaptic membrane. Upon the release of GABA from the presynaptic nerve terminal, the concentration of the transmitter in the synaptic cleft rapidly rises to millimolar concentrations (Grabauskas 2005; Scimemi and Beato 2009), activating the vast majority of the receptors in the postsynaptic membrane. The synaptic event is terminated by the removal of the transmitter by transporters and diffusion, followed by deactivation of the GABA_A_ receptor. The concentration of ambient GABA between synaptic events is near 1 *μ*mol/L (Lerma et al. 1986), to which the synaptic receptors are considered to be unresponsive due to their low resting affinity to the transmitter (Li and Akk 2015; Shin et al. 2017). Under physiological conditions, the receptor is also tonically exposed to the endogenous sulfonic acid taurine, and a number of potentiating (e.g., allopregnanolone) and inhibitory (e.g., pregnenolone sulfate) neurosteroids.

Coapplication of an allosteric agonist, for example, propofol or a neuroactive steroid, increases the current response to a low concentration of GABA. The effect manifests both as an augmented peak response and elevated steady‐state current amplitude (Li and Akk 2015; Germann et al. 2019). We previously showed that in human embryonic kidney cells expressing *α*1*β*2*γ*2L GABA_A_ receptors, the combination of clinical concentrations of propofol or etomidate with submicromolar concentrations of GABA generates steady‐state currents that are up to 10% of the peak response to saturating GABA. We inferred that such a potentiation of tonically activated synaptic‐type receptors contributes to the mechanisms of action of intravenous anesthetics on the GABA_A_ receptor (Li and Akk 2015).

**Figure 1:**
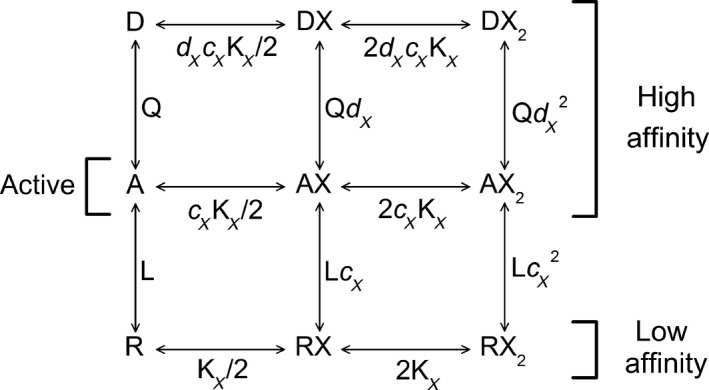
Resting‐Active‐Desensitized (RAD) model. The kinetic scheme is shown with two binding sites for agonist X. The receptor can occupy a resting (R), active (A), or desensitized (D) state. The active and desensitized states have high affinity to the agonist while the resting state has low affinity to the agonist. The parameter L (=R/A) describes the equilibrium between the resting and active states, and the parameter Q (=A/D) describes the equilibrium between the active and desensitized states. The parameter K_X_ is the equilibrium dissociation constant for X in the resting receptor. The parameter *c*_X_K_X_ is the equilibrium dissociation constant for X in the active receptor, and the parameter *d*_X_ *c*_X_K_X_ is the equilibrium dissociation constant for X in the desensitized receptor.

The goal of the present study was to quantitatively describe steady‐state activity of the *α*1*β*2*γ*2L GABA_A_ receptor in the presence of GABA, taurine, major endogenous neurosteroids, and the anesthetic propofol. Quantitative analyses and predictions of receptor activity were made in the framework of the three‐state cyclic Resting‐Active‐Desensitized model (termed: the RAD model; Fig. 1) (Germann et al. 2019). The data indicate that the steady‐state open probability (P_Open,S.S._) of the synaptic‐type *α*1*β*2*γ*2L receptor is ~0.008 under control conditions, that is, when exposed to ambient (1* μ*mol/L) GABA, and physiological levels of taurine (10 *μ*mol/L), and the steroids allopregnanolone (3*α*5*α*P; 10 nmol/L) and pregnenolone sulfate (PS; 0.1 *μ*mol/L). Exposure to clinical concentrations (0.3–1 *μ*mol/L) of propofol enhanced the P_Open,S.S._ by 2–4 fold. The findings are discussed in the context of total charge transfer during phasic and tonic transmission by the synaptic GABA_A_ receptor.

## Materials and Methods

### Receptors and expression

The GABA_A_ receptors were expressed in oocytes harvested from the African clawed frog (*Xenopus laevis*). The frogs were purchased from Xenopus 1 (Dexter, MI). Harvesting of oocytes was conducted under the Guide for the Care and Use of Laboratory Animals as adopted and promulgated by the National Institutes of Health. The animal protocol was approved by the Animal Studies Committee of Washington University in St. Louis (Approval No. 20170071).

The receptors consisted of rat *α*1*β*2*γ*2L subunits. The cDNAs in the pcDNA3 vector were linearized with XbaI (NEB Labs, Ipswich, MA), and the cRNAs were generated using mMessage mMachine (Ambion, Austin, TX). The oocytes were injected with a total of 3.5 ng cRNA in a 1:1:5 (*α*:*β*:*γ*) ratio. Following injection, the oocytes were kept in bath solution (96 mmol/L NaCl, 2 mmol/L KCl, 1.8 mmol/L CaCl_2_, 1 mmol/L MgCl_2_, 5 mmol/L HEPES; pH 7.4) with supplements (2.5 mmol/L Na pyruvate, 100 U/mL penicillin, 100 *μ*g/mL streptomycin, 50 *μ*g/mL gentamycin) at 16°C for 1–2 days prior to conducting electrophysiological recordings.

### Electrophysiology

The electrophysiological experiments were conducted at room temperature using two‐electrode voltage clamp. The oocytes were placed in the recording chamber (RC‐1Z, Warner Instruments, Hamden, CT) and clamped at −60 mV. Solutions were gravity‐applied, at a rate of 5–8 mL/min, from 30‐mL glass syringes with glass luer slips via Teflon tubing. Solutions were switched manually.

The currents were amplified with an Axoclamp 900A (Molecular Devices, Sunnyvale, CA) or OC‐725C amplifier (Warner Instruments, Hamden, CT), digitized with a Digidata 1320 or 1200 series digitizer (Molecular Devices), and stored on a PC using pClamp (Molecular Devices).

Drug applications to measure steady‐state activity lasted for 60–330 sec (1–5.5 min). Each cell was also tested with a brief application of 1 mmol/L GABA + 50 *μ*mol/L propofol that was considered to generate a peak response with open probability (P_Open,Peak_) indistinguishable from 1 (Shin et al. 2017).

### Data analysis

The current traces were analyzed using Clampfit (Molecular Devices) to determine the peak and steady‐state amplitudes. In cases where the current response had not reached steady‐state by the end of the agonist application (ΔI > 2% during the last 20 sec of agonist application), steady‐state current was estimated by exponential fitting of the current decay.

The conversion of raw current amplitudes to units of open probability has been described previously in detail (Forman and Stewart 2012; Eaton et al. 2016). In brief, the peak and steady‐state responses were matched against a scale ranging from P_Open_ of 0 (in the absence of GABAergic agents) to 1 (peak response in the presence of 1 mmol/L GABA + 50 *μ*mol/L propofol in the same cell). Curve fitting was done using Origin (OriginLab, Northampton, MA). Data are presented as mean ± SD.

### Predictions for current responses

The predictions for peak current responses were made as described in detail previously (Shin et al. 2019). In brief, the P_Open,Peak_ was calculated using the state function that pertains to the two‐state MWC model (Forman 2012; Steinbach and Akk 2019). The activation parameters (receptor affinity and efficacy) were taken from earlier studies (Shin et al. 2017; Akk et al. 2018; Shin et al. 2018). The predictions for steady‐state open probability were made using equation 1 (see below) that pertains to the three‐state model that incorporates a desensitized state (Fig. 1). In both models, the potentiating effect resulting from coapplying two or more agonists results from each agonist independently and additively contributing free energy toward stabilizing the active state.

When predicting responses to two or more agonists, the nominal concentration of each agonist was adjusted to account for cell‐to‐cell variability (Shin et al. 2019). By matching the experimental peak response to the previously determined concentration‐response relationship, the predicted response to a combination of agonists is based on the observed P_Open_ of responses to individual agonists rather than the nominal concentrations of the individual agonists. In other words, the predicted response to, for example, GABA + propofol is calculated based on the observed responses to GABA and propofol applied separately, rather than on the nominal concentrations of GABA and propofol.

### Materials and chemicals

The inorganic salts and HEPES used to prepare the bath solution were purchased from Sigma‐Aldrich (St. Louis, MO). GABA was purchased from Sigma‐Aldrich, solubilized in bath solution at 500 mmol/L, and stored in aliquots at −20°C. Taurine was purchased from Sigma‐Aldrich, solubilized in bath solution at 300 mmol/L, pH‐adjusted, and diluted as needed. Propofol was purchased from MP Biomedicals (Solon, OH). The stock solution of propofol was made in DMSO at 200 mmol/L and stored at room temperature. The steroid 3*α*5*α*P was bought from Sigma‐Aldrich or Tocris (Bio‐Techne, Minneapolis, MN), dissolved in DMSO at 10–20 mmol/L and stored at room temperature.

## Results

### Basic description of the three‐state model

The electrophysiological data were analyzed in the framework of the three‐state Resting‐Active‐Desensitized (RAD; Fig. 1) model. In this model, receptor behavior is described by six parameters. The constants L and Q are intrinsic to the receptor and describe the equilibrium between resting and active, and active and desensitized states, respectively. The parameters N_X_, K_X_, *c*_X_, and *d*_X_ are specific to the agonist. N_X_ is the number of binding sites for agonist X, and K_X_ the equilibrium dissociation constant of the resting receptor to agonist X. The parameters *c*_X_ and *d*_X_ give ratios of the equilibrium dissociation constants; *c*_X_ is the ratio of the constant for the active receptor relative to that for the resting receptor and *d*_X_ that of the desensitized receptor relative to that for the active receptor. A value of *c*_X_ or *d*_X_ < 1 indicates that the affinity is higher (lower dissociation constant) and accordingly that the binding of X tends to stabilize the A state relative to R or D relative to A.

The probability of being active in the framework of the RAD model in the presence of drug X is described by the following equation:

(1) $$P_{A,[X]}=\frac{1}{1+\frac{1}{Q}\left[\frac{1}{{\left(\frac{1+\left[X\right]/\left(c_{X}K_{X}\right)}{1+\left[X\right]/(d_{X}c_{X}K_{X}}\right)}^{N_{X}}}\right]+L{\left[\frac{1+\left[X\right]/K_{X}}{1+\left[X\right]/\left(c_{X}K_{X}\right)}\right]}^{N_{X}}}=\frac{1}{1+\frac{1}{Q\Delta_{\left[X\right]}}+L\Gamma_{\left[X\right]}}$$

where for simplicity

(2) $$\Gamma_{\left[X\right]}={\left[\frac{1+\left[X\right]/K_{X}}{1+\left[X\right]/\left(c_{X}K_{X}\right)}\right]}^{N_{X}}$$

and

(3) $$\Delta_{\left[X\right]}={\left[\frac{1+\left[X\right]/\left(c_{X}K_{X}\right)}{1+\left[X\right]/\left(d_{X}c_{X}K_{X}\right)}\right]}^{N_{X}}$$

We note that the probability of being in the active state (P_A_) is considered to be equal to the experimentally determined parameter P_Open_.

As indicated in Figure 1, the same sites are involved in all actions of X. If *c*_X_ = 1 then *Γ*_[X]_ is also 1 (i.e., the presence of X has no effect on the ratio of R to A states relative to the ratio in the absence of X), while if *d*_X_ = 1 then Δ_[X]_ = 1 (the presence of X does not alter the ratio of A to D).

For receptors which show minimal desensitization, that is, D/A → 0 and Q→∞, equation 1 approaches:

(4) $$P_{A,[X]}=\frac{1}{1+L\Gamma_{\left[X\right]}}$$

Equation 4 can be used to describe peak currents, assuming that desensitization develops slowly compared to development of peak response (Forman 2012; Steinbach and Akk 2019).

For high concentrations of efficacious agonists (*c*_X_<<1) or agonist combinations, the term L*Γ*_X_ in equation 1 becomes very small and P_A_ for steady‐state current approaches Q/(1 + Q):

(5) $$P_{A,\infty }=\frac{1}{1+\frac{1}{Qd_{X}^{N_{X}}}+L\Gamma_{\left[X\right]}}\to \frac{Qd_{X}^{N_{X}}}{1+Qd_{X}^{N_{X}}}$$

Accordingly, relative desensitization is predicted to be constant in the presence of saturating concentrations of all strong agonists or agonist combinations so long as the values for *d* are the same.

For two agonists, X and Y, interacting with distinct sites (e.g., GABA and propofol), equation 1 is modified as follows:

(6) $$P_{A,[X],[Y]}=\frac{1}{1+\frac{1}{Q\Delta_{\left[X\right]}\Delta_{\left[Y\right]}}+L\Gamma_{\left[X\right]}\Gamma_{\left[Y\right]}}$$

The denominator in equation 6 can be modified to add more *Γ* and Δ terms for additional agonists, as long as the additional agonists each interact with distinct sites.

When the agonists X and Y bind to the same sites (e.g., GABA and taurine), there are no longer separate and multiplicative terms for *Γ* and Δ. In this case, the probability that the receptor is active is:

(7) $$P_{A,[X],[Y]}=\frac{1}{1+\frac{1}{Q\Psi_{\left[X],[Y\right]}}+L\Omega_{\left[X],[Y\right]}}$$

where

(8) $$\Psi_{\left[X],[Y\right]}={\left[\frac{\left(1+\left[X\right]/c_{X}K_{X}+\left[Y\right]/c_{Y}K_{Y}\right)}{\left(1+\left[X\right]/\left(d_{X}c_{X}K_{X}\right)+\left[Y\right]/\left(d_{Y}c_{Y}K_{Y}\right)\right)}\right]}^{N}$$

and

(9) $$\Omega_{\left[X],[Y\right]}={\left[\frac{\left(1+\left[X\right]/K_{X}+\left[Y\right]/K_{Y}\right)}{\left(1+\left[X\right]/\left(c_{X}K_{X}\right)+\left[Y\right]/\left(c_{Y}K_{Y}\right)\right)}\right]}^{N}$$

In the most general sense, the receptor may be exposed to a combination of drugs acting at distinct or overlapping sites, with different affinities for the R, A, or D states. In this case, the overall level of activation would be described as follows:

(10) $$P_{A}=\frac{1}{1+\frac{1}{Q\prod_{X}\Delta_{\left[X\right]}\prod_{X,Y}\Psi_{\left[X],[Y\right]}}+L\prod_{X}\Gamma_{\left[X\right]}\prod_{X,Y}\Omega_{\left[X][Y\right]}}$$

The product symbols (Π) indicate that the product is taken over all drugs which bind to unique sites (X) or pairs of drugs (indicated by X, Y) which bind to the same overlapping sites, where the individual terms have been defined earlier.

The relatively simple form of the equation for a complicated combination of agonists and antagonists reflects the facts that in this model, different drugs do not interact with each other, and that the receptor changes state as a unit. Accordingly, the net result of the action of the many possible agents arises simply from the relative affinities of drugs for particular states of the receptor that result in stabilization of one state or another.

### Activation and desensitization properties of the recombinant *α*1*β*2*γ*2L GABA_A_ receptor

We exposed cells expressing *α*1*β*2*γ*2L receptors to 60–330 sec applications of 0.1–1000 *μ*mol/L GABA. Each cell was exposed to only 1–3 test concentrations of GABA to minimize measurement errors resulting from long‐duration recordings. For reference response, each cell was also exposed to 1 mmol/L GABA + 50 *μ*mol/L propofol that generated a peak response with open probability near 1 (Ruesch et al. 2012; Shin et al. 2017).

The activation parameters (K_GABA_, *c*_GABA_) were determined from the analysis of peak currents from 5 to 8 cells per agonist concentration using equation (4). With the number of GABA binding sites constrained to 2 (Amin and Weiss 1993) and L held at 8000 (Shin et al. 2017), we estimate a K_GABA_ of 16 ± 3 *μ*mol/L (best‐fit parameter ± SE of the fit) and a *c*_GABA_ of 0.0042 ± 0.0003. These estimates are similar to those reported previously (Chang and Weiss 1999; Rusch et al. 2004; Shin et al. 2017).

**Figure 2:**
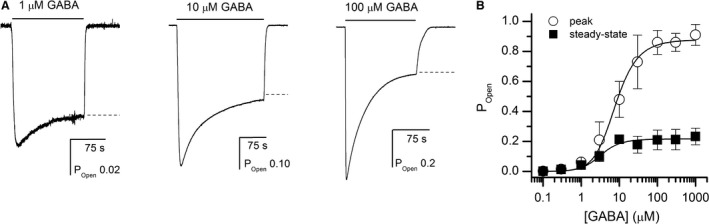
Peak and steady‐state activation of the *α*1*β*2*γ*2L receptor by GABA. Panel A shows sample current traces for receptors activated by 1, 10, or 100 *μ*mol/L GABA. The dashed lines show the steady‐state current levels as determined by exponential fitting of the decay phase. Panel B shows the concentration‐response relationships for peak and steady‐state currents. The data points show mean ± SD from 5 to 8 cells per concentration. The curves were fitted with equation 4 (peak data) and 1 (steady‐state data). The best‐fit parameters for peak currents are: K_GABA_ = 16 ± 3 *μ*mol/L and *c*_GABA_ = 0.0042 ± 0.0003. The curve for steady‐state currents was fitted using the K_GABA_ and c_GABA_ values constrained to those obtained in fitting the peak currents. The best‐fit value for *Q* was 0.29 ± 0.02. The value of L was constrained to 8000 (Shin et al. 2017), the number of binding sites for GABA was constrained to 2 (Amin and Weiss 1993), and the value of *d*_GABA_ was assumed to be 1 (see text for discussion).

In the second step of analysis, we fitted the concentration‐response relationship for steady‐state currents from the same set of cells to equation 1. The values of K_GABA_ and *c*_GABA_ were constrained to those determined in the analysis of peak responses (16 *μ*mol/L and 0.0042, respectively). N_GABA_ and L were held at 2 and 8000, respectively, as described above. Curve‐fitting the concentration‐response data yielded a Q (=A/D) of 0.29 ± 0.02. Thus, under steady‐state conditions, the ratio of active to desensitized receptors is ~ 1:3 for the *α*1*β*2*γ*2L receptor. Sample current responses and the GABA concentration‐response data for peak and steady‐state currents are shown in Figure 2.

**Figure 3:**
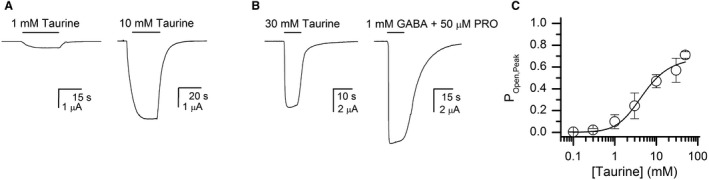
Peak activation of the *α*1*β*2*γ*2L receptor by taurine. Panel A shows sample current traces for receptors activated by 0.1 or 10 mmol/L taurine. Both traces are from the same cell. Panel B shows responses to 30 mmol/L taurine and 1 mmol/L GABA + 50 *μ*mol/L propofol (P_open _~1) from the same cell. Panel C shows the taurine concentration‐response relationship for peak currents. The curve was fitted with equation 4(3), yielding estimates for K_taurine_ of 5.1 ± 1.2 mmol/L and for *c*_taurine_ of 0.0075 ± 0.0006. The value of L was constrained to 8000 and the number of binding sites for taurine was constrained to 2.

Taurine is an endogenous sulfonic acid that has been implicated in modulating inhibitory transmission in the brain (Jia et al. 2008; Kletke et al. 2013). We determined the activation properties of taurine on the *α*1*β*2*γ*2L receptor. Cells expressing the *α*1*β*2*γ*2L receptor were exposed to 0.1–50 mmol/L taurine. The peak amplitudes were converted to P_Open_ units, and the concentration‐response curves fitted using equation 4. Curve‐fitting yielded a K_taurine_ of 5.1 ± 1.2 mmol/L, and a *c*_taurine_ of 0.0075 ± 0.0006. The number of binding sites for taurine was constrained to 2. Sample current traces and the concentration‐response relationship are shown in Figure 3.

We measured receptor desensitization in the presence of 30 mmol/L taurine, which is a saturating concentration. In five cells, the mean P_Open_ of the steady‐state response was 0.21 ± 0.05. The calculated (eq. 5) value of Q was 0.34 ± 0.14. We infer that receptor desensitization is fundamentally similar in the presence of GABA and taurine. We have assumed that *d*_GABA_ = 1. If this is not the case, then the value for Q is actually the inherent value for Q mutiplied by *d*_GABA_^2^.

### Inhibition of steady‐state current by the steroid pregnenolone sulfate

We next investigated receptor modulation by the endogenous steroid PS. Previous work has indicated that the steroid inhibits steady‐state activity by promoting entry to the desensitized state (Akk et al. 2001; Eisenman et al. 2003; Germann et al. 2019). Receptors were activated by 1 mmol/L GABA. Once the steady‐state current level was reached (typically following a 120–300 sec application), the cell was exposed to 1 mmol/L GABA plus 0.01–5 *μ*mol/L PS. Each cell was also tested with 1 mmol/L GABA + 50 *μ*mol/L propofol (P_open_ ~ 1). Application of PS alone did not result in any discernable activation, indicating that *c*_PS_ is very close to 1.

**Figure 4:**
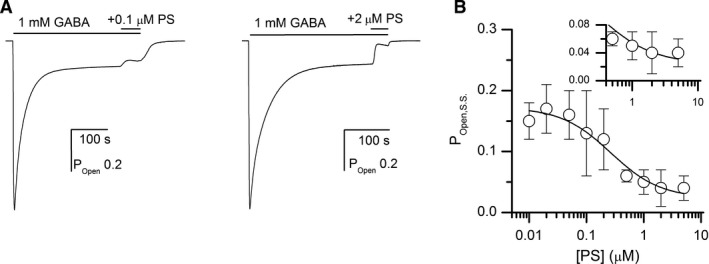
Receptor inhibition by the steroid pregnenolone sulfate. Panel A shows a sample current trace demonstrating the effect of 0.1 and 2 *μ*mol/L pregnenolone sulfate (PS) on steady‐state current from receptors activated by 1 mmol/L GABA. Panel B shows the PS concentration‐response relationship for receptors activated by 1 mmol/L GABA. The data points show mean ± SD from 4 to 5 cells per concentration. The curve was fitted with equation  (6), yielding a Q of 0.21 ± 0.02, K_PS_ of 1.9 ± 1.5 *μ*mol/L, and *d*_PS_ of 0.11 ± 0.06. The number of binding sites for PS was held at 1 and *c*_PS_ was assumed to be 1 (see text). The term L*Γ*_GABA_ was constrained to 0.08. The inset more clearly demonstrates the incomplete block at high concentrations of PS. Fitting the concentration‐response data to the Hill equation yielded an IC_50_ of 0.25 ± 0.05 *μ*mol/L.

Exposure to PS reduced the steady‐state current level. Fitting the concentration‐response data to the Hill equation yielded an IC_50_ of 0.25 ± 0.05 *μ*mol/L and a Hill coefficient of −1.86 ± 0.53. The PS concentration‐response data were also analyzed using equation 6. Curve‐fitting yielded a K_PS_ of 1.9 ± 1.5 *μ*mol/L, and a *d*_PS_ of 0.11 ± 0.06 with the number of PS binding sites constrained to 1. These are similar to the values (3.5 *μ*mol/L and 0.054, respectively) reported recently for the concatemeric *α*1*β*2*γ*2L GABA_A_ receptor (Germann et al. 2019). When N_PS_ was held at 2, the fitted K_PS_ was 1.0 ± 0.6 *μ*mol/L, and *d*_PS_ 0.35 ± 0.07. There was, however, no improvement in the quality of the fit (not shown). Sample currents and the PS concentration‐response relationship are shown in Figure 4.

### Propofol can increase the steady‐state activation of receptors by GABA

For efficacious agonists (or agonist combinations), the open probability of the peak response is near 1. The value of Q can then be directly estimated from the relative steady‐state response using equation 5. For receptors activated by 1 mmol/L GABA, the P_Open_ for steady‐state current was 0.18 ± 0.09 (n = 5 cells), and the calculated value of Q 0.24 ± 0.15 (assuming the value of L*Γ*_X_ in eq. 5 approaches 0). This is similar to the value of Q estimated from fitting the concentration‐response curve for steady‐state data (0.29).

To determine the effects of allosteric agonists on desensitization, we measured the effects of 3*α*5*α*P and propofol on steady‐state current. The experiments were conducted by first activating the receptors with 1 mmol/L GABA. Upon reaching the steady‐state response, the drug flow was switched to 1 mmol/L GABA + 1 *μ*mol/L 3*α*5*α*P or 1 mmol/L GABA + 10 *μ*mol/L propofol.

When the switch was made to GABA + 3*α*5*α*P, the level of steady‐state response remained unaffected. In 5 cells, the steady‐state current level following the switch to GABA + 3*α*5*α*P was 100 ± 1% of the current level in the presence of GABA alone. The lack of discernable change indicates that the value for *d*_3_ *_α_*_5_ *_α_*_P_ is very close to that for GABA, which we assume is unity. In contrast, a switch from GABA to GABA + propofol was accompanied by an increase in steady‐state current. The application of propofol increased the steady‐state current by 1.51 ± 0.21‐fold (n = 5 cells).

The peak P_Open_ in the presence of 1 mmol/L GABA is 0.91 (Fig. 2) whereas coapplication of propofol with saturating GABA increases the P_Open_ to near unity (Ruesch et al. 2012; Shin et al. 2017). Hence, coapplication of propofol is also expected to enhance P_Open,S.S._. However, this mechanism should only produce approximately a 10% increase in steady‐state P_Open_, rather than the 50% increase observed.

Alternatively, an increase in steady‐state current upon coapplication of propofol with GABA may result from propofol having higher affinity to the active than desensitized state of the receptor, thereby stabilizing the active receptor. To explore this possibility, we recorded the concentration‐response relationship for propofol‐induced enhancement of steady‐state current. A cell was initially exposed to 1 mmol/L GABA. Once the current response neared steady‐state, 0.05–10 *μ*mol/L propofol was coapplied with GABA. Each cell was exposed to only a single concentration of propofol.

**Figure 5:**
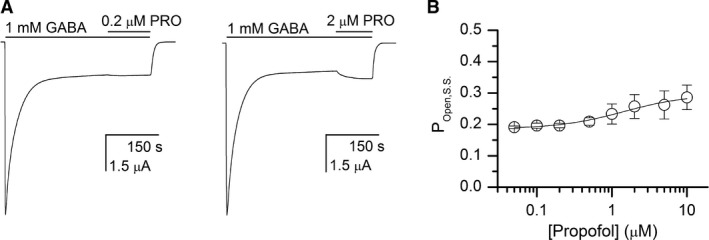
Modulation of steady‐state current by propofol. Panel A shows sample current traces for receptors activated by 1 mmol/L GABA and exposed to 0.2 or 2 *μ*mol/L propofol (PRO). Coapplication of propofol increases the steady‐state current level. Panel B shows the propofol concentration‐response relationship for modulation of steady‐state open probability in the presence of 1 mmol/L GABA. The data points show mean ± SD from 5 to 7 cells. The curve was fitted with equation 7, yielding a K_PRO_ of 1.2 ± 0.4 *μ*mol/L for the active state and a *d*_PRO_ (ratio of the equilibrium dissociation constants of the active and desensitized states) of 1.17 ± 0.01, with the number of binding sites constrained to 4. The fitted value of Q was 0.24 ± 0.01. The term L*Γ*_GABA_ was constrained to 0.08.

The propofol‐induced changes in steady‐state open probability were analyzed using equation 6. Curve‐fitting of the pooled data from 5 to 7 cells per concentration gave a K_PRO_ of 1.2 ± 0.4 *μ*mol/L for the active state and a *d*_PRO_ (ratio of the equilibrium dissociation constants of the desensitized and active states) of 1.17 ± 0.01, with the number of binding sites arbitrarily constrained to 4. Sample traces and the concentration‐response relationship are provided in Figure 5. We note that the RAD model explicitly includes the potentiation produced by propofol, by the inclusion of the *Γ*_PRO_ term modifying L.

We next tested the effects of the *β*2(Y143W) and *β*2(M286W) mutations on propofol‐induced enhancement of steady‐state current. These mutations reduce receptor activation by propofol likely by modifying propofol interactions with the individual binding sites on the receptor (Krasowski et al. 1998; Eaton et al. 2015; Shin et al. 2018; Szabo et al. 2019). We hypothesized that if these identified propofol interaction sites mediate the observed increase in P_Open,S.S._, then the mutations to the *β*2(Y143) and *β*2(M286) residues will reduce the effect.

The application of 10 *μ*mol/L propofol enhanced the steady‐state open probability in the presence of saturating GABA by 1.15 ± 0.10‐fold (n = 7 cells) or 1.16 ± 0.12‐fold in receptors containing the *β*2(Y143W) or *β*2(M286W) mutation, respectively. This is similar to the predicted (1.22‐fold) potentiation assuming that either *β*‐subunit mutation eliminates two equipotent and equiefficacious binding sites for propofol. We also tested the effect of propofol on steady‐state current in the receptor containing both mutations (*β*2(Y143W + M286W)). In this receptor, the current level in the presence of 10 *μ*mol/L propofol was reduced by 21 ± 12% (n = 5 cells). We do not have an explanation for why exposure to propofol reduces steady‐state current, although it is known that propofol also blocks channels (Adodra and Hales 1995; Ruesch et al. 2012; Shin et al. 2018). The contributions of increased maximal P_Open_ and possible block make the quantitative predictions of the effects of the mutations somewhat uncertain. However, the overall data on mutated receptors support the idea that the increase in steady‐state P_Open_ in the presence of propofol is mediated by previously identified sites that are also involved in activation and potentiation. This conclusion is consistent with the RAD model.

### Steady‐state current in the presence of agonist combinations

To test the generality of the RAD model in predicting steady‐state activity and to determine the independence of actions of orthosteric and allosteric agonists, we recorded current responses from cells exposed to several agonist combinations and combinations of concentrations. The experimentally observed responses were compared with those predicted using equation 10. We note that in these experiments, the concentrations of individual agonists were selected to produce a wide range of steady‐state open probability with no regard to physiological levels of the respective ligands. The concentration of GABA ranged from 0.5 *μ*mol/L to 1 mmol/L, propofol from 2 to 10 *μ*mol/L, 3*α*5*α*P from 0.1 to 1 *μ*mol/L, and PS from 0.5 to 1 *μ*mol/L. In all, 14 combinations of drugs and drug concentrations were tested.

**Figure 6:**
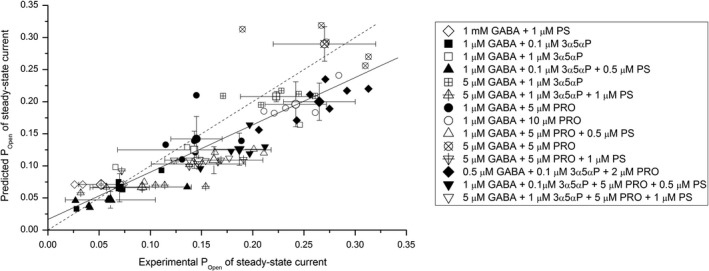
Steady‐state activation of the *α*1*β*2*γ*2L receptor by combinations of agonists. The graph shows the observed and predicted P_Open_ of steady‐state responses in the presence of GABA and various combinations of allopregnanolone (3*α*5*α*P), pregnenolone sulfate (PS), and propofol (PRO). The predicted values were determined using equation  (10) modified to reflect the agonistic effects of GABA, 3*α*5*α*P, and propofol, and the effects of PS and propofol on the equilibrium between Active and Desensitized states. The small symbols show data from individual cells. The large symbols show mean ± SD for each agonist combination. The solid line gives the linear fit to all data points (*R* ^2^ = 0.85, *P* < 0.0001). The dashed line shows ideal agreement between predicted and experimental P_Open_.

Overall, there is a good agreement between observed and predicted P_Open,S.S._ values. A global fit of all 72 data points to linear regression yielded an *R* ^2^ of 0.85 (*P* < 0.0001) with a regression slope close to 1 (0.76 ± 0.06). The findings are presented in Figure 6.

### Simulations of tonic activation of the synaptic GABA_A_ receptor

We used equation 10 to generate predictions of synaptic receptor activity under steady‐state conditions, that is, in the absence of phasic, presynaptic GABA release. The predictions were made assuming that the receptor is exposed to GABA, taurine, 3*α*5*α*P, and PS. Additionally, we simulated the effect of propofol on receptor activity to mimic exposure during clinical anesthesia. For all predictions, the concentration of GABA was held at 1 *μ*mol/L (Lerma et al. 1986), and the concentration of taurine at 10 *μ*mol/L (Lerma et al. 1986; Molchanova et al. 2004). The concentration of PS was constrained to 0.1 *μ*mol/L (de Peretti and Mappus 1983; Weill‐Engerer et al. 2002). The concentration of 3*α*5*α*P varied between 1 nmol/L and 10 *μ*mol/L (this large range was used in order to demonstrate saturation of the steroid concentration‐response relationship). The simulations were made in the absence of propofol and in the presence of 0.1, 0.3, or 1 *μ*mol/L propofol (Engdahl et al. 1998; Dawidowicz et al. 2003).

**Figure 7:**
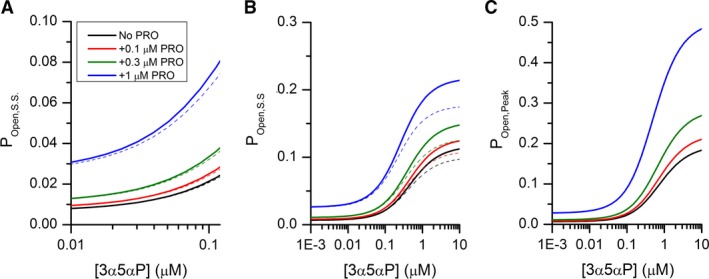
Predicted steady‐state and peak currents in the presence of combinations of GABAergic drugs. Panels A and B show steady‐state currents predicted using equation 10. In all simulations, the *α*1*β*2*γ*2L receptor was exposed to 1 *μ*mol/L GABA and 10 *μ*mol/L taurine, whereas the concentration of 3*α*5*α*P varied between 0.01 and 0.1 *μ*mol/L (Panel A), or 1 nmol/L and 10 *μ*mol/L (Panel B) to illustrate the full concentration‐response relationship. The simulations were done in the absence of propofol (PRO; black line) or in the presence of 0.1 (red line), 0.3 (green line), or 1 *μ*mol/L propofol (blue line). The effect of coapplication of 0.1 *μ*mol/L PS on steady‐state current is shown as dashed lines, color‐coded for the presence of propofol. Panel C shows the predicted peak currents in the presence of 1 *μ*mol/L GABA and 10 *μ*mol/L taurine combined with 1 nmol/L–10 *μ*mol/L 3*α*5*α*P and 0–1 *μ*mol/L propofol.

A graphic presentation of steady‐state P_Open_ is provided in Figure 7. The major finding is that coapplication of propofol strongly increases P_Open,S.S._. Exposure to 1 *μ*mol/L propofol had an almost fourfold effect at physiological concentrations (0.01–0.1 *μ*mol/L) of 3*α*5*α*P (Fig. 7A). Coapplication of 0.1 *μ*mol/L PS had a relatively minor effect on P_Open,S.S._, largely due to its low affinity. A change in the concentration of 3*α*5*α*P had a relatively small effect on steady‐state current. A tenfold increase in 3*α*5*α*P concentration, from 0.01 to 0.1 *μ*mol/L, approximately doubled the P_Open,S.S._, both in the absence and presence of propofol.

## Discussion

While synaptic GABA_A_ receptors are most conspicuously activated by phasically released GABA from presynaptic nerve terminals, the receptors are exposed, persistently, to a number of endogenous agonists such as taurine and neuroactive steroids. Administration of clinical agents, including GABAergic anxiolytics and sedatives, is also characterized by relatively long exposure times. This raises a possibility that continuous exposure to combinations of orthosteric and/or allosteric agonists leads to meaningful tonic activation of the synaptic GABA_A_ receptor. The goal of this study was to quantitatively describe steady‐state activity in the synaptic‐type *α*1*β*2*γ*2L GABA_A_ receptor. The receptors were activated by combinations of GABA, taurine, 3*α*5*α*P, PS, and propofol. Analysis and predictions of receptor activity were done in the framework of a three‐state Resting‐Active‐Desensitized (RAD) model (Fig. 1; (Germann et al. 2019)). We infer that tonic activity due to exposure to ambient GABA, taurine, and neuroactive steroids contributes significantly to overall function of the synaptic GABA_A_ receptor.

We began by investigating the desensitization properties of the *α*1*β*2*γ*2L receptor. In the RAD model, the extent of desensitization is determined by the equilibrium constant between the active and desensitized states (termed Q; Fig. 1). Analysis of peak and steady‐state currents in the presence of GABA yielded an estimate for Q of 0.29, indicating that under steady‐state conditions the ratio of receptors in the active vs. desensitized state is ~1:3. Using equation 5, we estimate that the maximal open probability for steady‐state activity is 0.23 in the *α*1*β*2*γ*2L receptor in the presence of GABA. Coapplication of 3*α*5*α*P with GABA did not significantly modify steady‐state current, whereas exposure to PS and propofol decreased and increased, respectively, the P_Open,S.S._. Analysis of these effects using equation 6, in which a change in P_Open,S.S._ is mediated by different affinities of the drug to active and desensitized states, yielded a ratio of equilibrium dissociation constants of active versus desensitized state of 2.86 for PS and 0.85 for propofol. In contrast to these allosteric agents, the orthosteric agonist taurine and allosteric agonist 3*α*5*α*P appear to be similar to GABA in that they have indistinguishable affinities for the active and desensitized states.

Throughout the analysis we have assumed that GABA binds with identical affinities to active and desensitized receptors (i.e., *d*_GABA_ = 1). This assumption is based on detailed single‐channel analysis of the related nicotinic acetylcholine receptor for which it was shown that the transmitter ACh binds with similar affinities to the active and desensitized states (Sine et al. 1995; Grosman and Auerbach 2001). Strictly, our data show that the values of *d* are the same in the presence of GABA and taurine, while *d*_PS_ is less and *d*_PRO_ is greater than *d*_GABA_.

Coapplication of multiple agonists interacting with distinct sites increases the peak response through energetic additivity where each agonist independently modifies the effective value of L (Fig. 1; Ruesch et al. 2012; Akk et al. 2018; Shin et al. 2019)). To test whether the principle of energetic additivity applies to steady‐state currents in the presence of multiple active agents, we compared the observed P_Open,S.S._ recorded in the presence of several agonist combinations and concentrations of individual agonists with those calculated using equation 10. The data summarized in Figure 6 indicate that there is a good agreement between experimental observations and simulations for a wide range of P_Open,S.S._.

The RAD model is an extension of the initial MWC model. In the complete RAD model shown in Figure 1 there are three functional states and three affinity states. However, the essential features of the MWC model are retained – that the receptor changes state as a unit (so individual subunits of the receptor are all in the same state) and that all sites are identical. Under these conditions the actions of multiple agonists and antagonists all take place at the level of stabilizing one state or another. This is the most practically relevant aspect of this class of models: interactions among agents can be described by analyzing the actions of one agent in the absence of others, and the effects of combinations predicted without assuming that there are specific interactions between agents (i.e., that one drug affects the affinity of another). However, it is clear that the GABA_A_ receptor and other transmitter‐gated channels have more than one active and more than one desensitized state, and known drug actions are not included (for example open channel block). In addition, these models do not describe the kinetics of activation. The relative success of the model in describing receptor properties suggests that the states labelled R, A, and D reflect average energies of sets of states with same function.

To simulate steady‐state activity under physiological conditions, we assumed that the receptor is exposed to 1 *μ*mol/L GABA (Lerma et al. 1986), 10 *μ*mol/L taurine (Lerma et al. 1986; Molchanova et al. 2004), and 10–100 nmol/L 3*α*5*α*P (Cheney et al. 1995; Weill‐Engerer et al. 2002). The latter can be considered to reflect the net concentration of all species of potentiating steroids, for example, pregnanolone (3*α*5*β*P), tetrahydrodeoxycorticosterone (THDOC) and others, in addition to 3*α*5*α*P. The sum concentration of all potentiating steroids species is almost certainly higher than the concentration of 3*α*5*α*P employed in the calculations above. Because the steroids interact with the same set of sites, the effects are additive in terms of concentrations (Shin et al. 2019). For example, five potentiating steroid species with similar affinities and efficacies present at 10 nmol/L concentration each, effectively act as a single potentiating steroid at 50 nmol/L. We also assumed that the receptors are exposed to 0.1 *μ*mol/L PS (de Peretti and Mappus 1983; Weill‐Engerer et al. 2002). Again, this reflects the net concentration of all inhibitory steroids. Finally, we tested the effect of exposure to 0.1–1 *μ*mol/L propofol (Engdahl et al. 1998; Dawidowicz et al. 2003) on steady‐state activity.

Several conclusions can be made from the simulations (Fig. 7A). First, the *α*1*β*2*γ*2L receptor is minimally active with a P_Open,S.S._ of ~ 0.007 when exposed to ambient GABA and taurine, although this is greater than the P_Open_ in the absence of any agonist (constitutive P_Open_ = 0.00012; (Shin et al. 2017)). Second, exposure to endogenous potentiating steroids has a relatively small effect on receptor activity. The P_Open,S.S._ ranges from 0.01 to 0.02 when 10–100 nmol/L 3*α*5*α*P (or equivalent potentiating steroids) is coapplied with GABA and taurine (Fig. 7A). Exposure to 0.1 *μ*mol/L PS (or equivalent inhibitory steroids) is essentially without effect on GABA_A_ receptor activity (dashed lines Fig. 7A–B). Finally, propofol increases P_Open,S.S._. For example, in the presence of GABA, taurine, 10 nmol/L 3*α*5*α*P and 0.1 *μ*mol/L PS, exposure to 0.3 or 1 *μ*mol/L propofol increases P_Open,S.S._ from 0.008 to 0.013 or 0.03, respectively.

To put these P_Open,S.S._ values in perspective, we compared total charge transfer for phasic and tonic activity at a single synapse. For a 1 sec time period, the integrated charge transfer for tonic activity, in units of P_Open_ × s, is 0.008, 0.013, and 0.03 in the absence of propofol and in the presence of 0.3 or 1 *μ*mol/L propofol, respectively. The P_Open_ × s units can be readily converted to more conventional units. For example, by assuming that a synapse contains 100 activatable *α*1*β*2*γ*2L receptors, each generating 1 pA current, the charge transfer is 0.8 pA × s (0.8 pC) in the absence of propofol and 1.3 or 3 pA × s (1.3 or 3 pC) in the presence of 0.3 or 1 *μ*mol/L propofol.

For phasic activity, it is considered that during the peak synaptic response 100% of receptors are active, that is, P_Open,Peak_ is indistinguishable from 1 (Kitamura et al. 2003; Takahashi et al. 2006; McDougall et al. 2008). The decay time constants of sIPSCs are 30 msec under control conditions, and 65 or 90 msec in the presence of 0.3 or 1 *μ*mol/L propofol (Cao et al. 2018). Then, the total charge transfer for a single sIPSC is 0.03, 0.065, or 0.09 P_Open_ × s in the presence of 0, 0.3, or 1 *μ*mol/L propofol, respectively. In SI units, the total charge transferred during a single sIPSC is 3 pC in the absence of propofol and 6.5 pC and 9 pC in the presence of 0.3 and 1 *μ*mol/L propofol, respectively.

These calculations were made on a per synapse basis. The true relative charge transfer thus depends on the frequency of synaptic events at an average synapse. The reported total cellular frequency of sIPSCs in various preparations ranges from ~1 to >10 Hz (Hajos and Mody 1997; Browne et al. 2001; Chakrabarti et al. 2016). With 10–1000 synapses per cell (Ichikawa et al. 1993; Cullen et al. 2010; Zhao et al. 2016), the estimated frequency of sIPSCs per synapse thus varies between 0.001 and 1 Hz. Accordingly, the relative integrated charge transfer may range from 0.996:0.004 (at 0.001 Hz frequency per synapse) to 0.21:0.79 (at 1 Hz) for the ratio of tonic to phasic activity in the presence of ambient GABA and physiological taurine, 3*α*5*α*P, and PS. While exposure to propofol enhances the P_Open,S.S._ (Fig. 7) and increases the decay time constants of sIPSCs (Cao et al. 2018), its relative effects are similar on tonic and phasic activation of the synaptic‐type receptor and, therefore, on the ratios of relative integrated charge transfer (0.997:0.003 for tonic vs. phasic in the presence of 1 *μ*mol/L propofol at 0.001 Hz frequency per synapse and 0.25:0.75 at 1 Hz).

These ratios are independent of the assumptions made in calculating total charge transfer (i.e., 100 channels at 1 pA each), suggest that tonic activation of the synaptic GABA_A_ receptor is a significant, and under some conditions, the major contributor to overall charge transfer. We emphasize that this comparison strictly applies to synaptically located receptors. A pool of *α*1‐containing synaptic‐type receptors is located outside of the synapse (Thomas et al. 2005; Kasugai et al. 2010); these receptors presumably contribute only to tonic activity. Although the data indicate that phasic activity only partially contributes to overall charge transfer in the synaptic‐type GABA_A_ receptor, the true physiological significance and role of each type of activity remains to be determined. For example, it may be expected that the larger conductance associated with phasic synaptic transmission may have a larger effect on the probability a cell will fire an action potential when the IPSC is temporally associated with excitatory input. One implication of this is that while propofol similarly modifies tonic and phasic activity in the *α*1*β*2*γ*2 receptor, its clinical effects due to a decreased probability of action potential firing are likely mediated through phasic transmission.

Lastly, tonic inhibition has been associated with the *α*4*β*δ subtype in many brain regions. Our experiments did not address the relative contributions of *α*1*β*2*γ*2L and *α*4*β*δ receptors to overall tonic GABAergic activity. However, given that *α*4*β*δ receptors are constitutively active (e.g., (Tang et al. 2010)) and have larger open probability in the presence of submicromolar concentrations of GABA (e.g., (Eaton et al. 2014)), our data on steady‐state activation of the *α*1*β*2*γ*2L receptor corroborate that tonic inhibition in neurons is largely mediated by the *α*4*β*δ rather than the synaptic‐type *α*1*β*2*γ*2L receptor.

## Conflict of Interest

The authors declare that they have no competing interests.
